# A new on-axis multimode spectrometer for the macromolecular crystallography beamlines of the Swiss Light Source

**DOI:** 10.1107/S0909049508040120

**Published:** 2009-02-25

**Authors:** Robin L. Owen, Arwen R. Pearson, Alke Meents, Pirmin Boehler, Vincent Thominet, Clemens Schulze-Briese

**Affiliations:** a Swiss Light Source, Paul Scherrer Institute, CH-5232 Villigen PSI, Switzerland; b Astbury Centre for Structural Molecular Biology, Astbury Building, The University of Leeds, Leeds LS2 9JT, UK

**Keywords:** single-crystal microspectrophotometry, kinetic crystallography, structural enzymology, radiation damage, macromolecular crystallography, complementary techniques

## Abstract

X-ray crystallography at third-generation synchrotron sources permits tremendous insight into the three-dimensional structure of macromolecules. Additional information is, however, often required to aid the transition from structure to function. *In situ* spectroscopic methods such as UV–Vis absorption and (resonance) Raman can provide this, and can also provide a means of detecting X-ray-induced changes. Here, preliminary results are introduced from an on-axis UV–Vis absorption and Raman multimode spectrometer currently being integrated into the beamline environment at X10SA of the Swiss Light Source. The continuing development of the spectrometer is also outlined.

## Abstract 2

Complementary techniques greatly aid the interpretation of macromolecule structures to yield functional information, and can also help to track radiation-induced changes. A new on-axis spectrometer being integrated into the macromolecular crystallography beamlines of the Swiss Light Source is presented.

## 1. Introduction

In order to fully understand biological processes, a knowledge of the atomic structure of the macromolecules involved is essential. The most common method of structure determination is X-ray crystallography, and the many protein and DNA structures already determined have provided incredible insights into the molecular underpinnings of life. Most X-ray crystal structures, however, are static average snapshots of a molecule and only yield information about a single state of a complex reaction. In addition, the deleterious effects of radiation damage can cast uncertainty on the validity of the model itself. More information about function can be obtained *via* kinetic crystallography, and one form this can take is soaking experiments. These take advantage of the fact that, as long as the active site is not blocked by crystal packing, and turnover does not require any major structural rearrangements that could disrupt the crystalline lattice, many enzymes retain their catalytic activity in the crystalline state. By soaking in a substrate and flash-cooling the crystal in liquid nitrogen, intermediate species can be freeze-trapped for structure determination (Kovaleva & Lipscomb, 2007 ▶; Schlichting & Chu, 2000 ▶; Katona *et al.*, 2007 ▶). Identification of the trapped species, however, is limited by the resolution of the diffraction data obtained and the occupancy of the trapped state. This is often insufficient to unambiguously assign the chemical intermediate observed in the electron density map, especially in cases in which there are no large conformational changes in the ligand and/or protein. This ambiguity highlights the need for spectroscopic methods complementary to X-ray crystallography in structural biology to identify the exact chemical state of the trapped species.

The most commonly accessed of these methods to date is single-crystal UV/visible spectroscopy (UV-SCS) (Pearson *et al.*, 2004 ▶), although fluorescence (Royant *et al.*, 2007 ▶; Bourgeois *et al.*, 2002 ▶; Klink *et al.*, 2006 ▶), Raman (Carpentier *et al.*, 2007 ▶) and Fourier-transform infrared (Moukhametzianov *et al.*, 2006 ▶) spectroscopy are also in use. Ideally, spectra are recorded at 100 K from the same crystal used for X-ray diffraction data collection, though this is not always possible. As a result of the increasing interest in this field, several single-crystal microspectrophotometers are now available worldwide at both synchrotron sources and home laboratories; for a recent review see De la Mora-Rey & Wilmot (2007 ▶). The synchrotron systems in particular are designed to operate both on- and off-line, allowing direct monitoring of crystal spectra during X-ray data collection.

As well as the ability to identify trapped reaction intermediates, on-line microspectrophotometers have also allowed the monitoring of the effects of X-ray exposure upon protein crystals. It is well established that excessive X-ray exposure results in eventual loss of diffraction signal (Henderson, 1990 ▶; Owen *et al.*, 2006 ▶). However, both on-line single-crystal spectroscopy and X-ray absorption near-edge spectroscopy have revealed that chemical and conformational changes can occur in the crystal at much lower doses than are usually considered to cause radiation damage (Beitlich *et al.*, 2007 ▶; Yano *et al.*, 2005 ▶).

**Figure 1:**
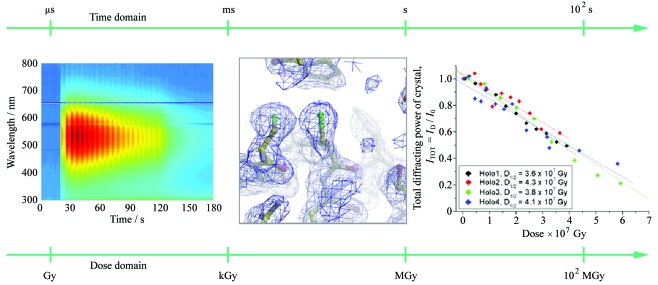
Manifestations of radiation damage over a wide range of time scales: UV–Vis absorption spectra of irradiated ethylene glycol [data collected at the ESRF using the arrangement described by Southworth-Davies & Garman (2007 ▶)] showing a rapid increase in absorption owing to 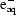 upon X-ray irradiation; disulphide bond breakage in low-dose composite data sets of DsbA (R. L. Owen, unpublished data); decay of diffracting power of holoferritin crystals as a function of X-ray dose (Owen *et al.*, 2006 ▶).

These changes are illustrated in Fig. 1 ▶ and range from a near instantaneous generation of aqueous, or solvated, electrons (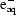) upon X-ray exposure, through fast changes at electron-sensitive centres such as redox sites or disulphide bonds at low X-ray dose, to the eventual decay of diffraction and crystal death. These radiation-induced changes have been observed in a variety of biological systems (for example, see Berglund *et al.*, 2002 ▶; Adam *et al.*, 2004 ▶; Hough *et al.*, 2008 ▶; Pearson *et al.*, 2007 ▶).

Unfortunately only a small subset of proteins have functional groups amenable to UV–Vis absorption spectroscopy; additional complementary methods are therefore required for tracking X-ray-induced changes or obtaining electronic and vibrational information from crystals. Raman spectroscopy has the advantage of not requiring coloured functional groups, and can provide detailed information (Carey, 1999 ▶; Hilde­brandt & Lecomte, 2000 ▶; Tuma, 2005 ▶). Raman spectroscopy is analogous to infrared absorption spectroscopy in that it probes the vibrational energy levels of molecules rather than the electronic transitions probed by UV–Vis absorption.

Vibrational spectroscopy provides information with a sensitivity beyond that usually achieved in a macromolecular diffraction experiment. Information on protonation states, van der Waals and electrostatic interactions can be directly measured rather than inferred, greatly facilitating the transition from structure to function (for an overview, see Siebert & Hildebrandt, 2007 ▶). For a multimode spectrometer targeting biological samples, infrared absorption has several disadvantages when compared with Raman spectroscopy. CCD detectors and lenses optimized for UV–Vis absorption are non-ideal for IR and *vice versa*, and therefore for multimode spectroscopy UV–Vis and Raman spectroscopies are more complementary. Infrared absorption is also limited by the so-called ‘water problem’: water has strong absorption bands in the infrared limiting the possible sample size and environment (Susi, 1969 ▶). While macromolecular Raman spectroscopy poses several challenges, primarily in the form of the extremely weak nature of the effect, obscuration of bands by fluorescence and difficulties in interpreting spectra, Raman spectroscopy nonetheless provides a rich source of complementary information for crystallographers and is gaining in popularity (Carey, 2006 ▶; Katona *et al.*, 2007 ▶). Processes involving changes within a crystal, for example kinematic and ligand binding experiments, are greatly simplified by use of difference Raman spectroscopy easing difficulties in interpretation of spectra. Protein fluorescence can also be a valuable source of information on changes in local environment and conformation during X-ray data collection, and the prospects of using the microspectrophotometer described here for this and other spectroscopies will be outlined in §4.

Some of the challenges associated with Raman spectroscopy mentioned above can be bypassed by exploiting resonance effects. Resonance Raman occurs when the frequency of the laser probe is tuned to that of an electronic transition in the molecule of interest. This highlights an area in which the UV–Vis absorption and Raman modes of a multimode spectrometer are complementary to each other, as well as to X-ray diffraction. The UV–Vis absorption spectrum of a molecule reveals the wavelengths at which Raman modes will be selectively enhanced under resonant conditions by a factor of up to 10^6^, allowing bands to be clearly discerned above fluorescence and specific regions of a molecule to be probed. This enhancement means that Raman data acquisition times can be reduced to match those in macromolecular crystallography, if a laser wavelength matching an electronic transition of the biological molecule is available. Good Raman data can also be collected under non-resonant conditions, although these require acquisition times of the order of several minutes.

The most commonly accessed biological resonant modes are exhibited for iron- or copper-containing proteins at an excitation wavelength of 413.1 nm (Kr^+^ gas laser probe), for example myoglobin, cytochrome C oxidase, rubredoxins and azurins (Coyle *et al.*, 2003 ▶; Konishi *et al.*, 2004 ▶; van Amsterdam *et al.*, 2002 ▶; Sanders-Loehr, 1988 ▶; Engler *et al.*, 2000 ▶; Averill *et al.*, 1987 ▶), or 514 nm (Ar^+^ gas laser probe), for example purple acid phosphatase and rubredoxin (Xiao *et al.*, 2005 ▶; Averill *et al.*, 1987 ▶). Some of these modes can also be accessed using longer laser wavelengths, for example cytochrome C oxidase at 580–615 nm (Bocian *et al.*, 1979 ▶; Czernuszewicz *et al.*, 1994 ▶) and rubredoxin at 647.1 nm (Czernuszewicz *et al.*, 1994 ▶). Increasing the range of available laser wavelengths correspondingly increases the range of accessible metal–ligand modes; red laser lines, for example, make possible resonant scattering from rhodopsin (600 nm) (Mathies *et al.*, 1976 ▶), methane monooxygenase (647.1 nm) (Liu *et al.*, 1995 ▶) and galactose oxidase (659 nm) (Whittaker *et al.*, 1989 ▶). Care must be taken, however, in resonance Raman experiments to avoid laser-induced changes (Tonge *et al.*, 1993 ▶; Meents *et al.*, 2007 ▶) as photochemical processes are enhanced when the laser wavelength matches an electronic transition (Turro, 1991 ▶). In the case of structural biology the primary advantage of moving to longer wavelengths is the accompanying decrease in protein fluorescence, so that non-resonance Raman bands are not obscured by this unwelcome effect (Carey, 2006 ▶).

Owing to the highly concentrated and spatially ordered nature of biological molecules in the crystalline state, Raman crystallography can provide superior spectra to solution phase spectroscopy. In particular, the small spectral changes associated with ligand binding are more readily followed (Carey & Dong, 2004 ▶; Altose *et al.*, 2001 ▶; Helfand *et al.*, 2003 ▶; and references above). As Raman scattering is a tensorial quantity (Tsuboi & Thomas, 1997 ▶), care must therefore be taken with alignment of the crystal axes of samples, since changes in orientation can cause the relative intensities of different bands to change. Raman spectroscopy has also recently been used to measure degradation of selenomethionine derivatives (Vergara *et al.*, 2008 ▶), providing further motivation for the implementation of an instrument for on-line Raman spectroscopy.

In summary, Raman and UV–Vis absorption spectroscopies can provide information complementary to that obtained using X-ray diffraction, and are well placed to greatly facilitate the development of a new temporal dimension in macromolecular crystallography. The inherent difference between Raman spectroscopy and UV–Vis absorption, in that inelastic scattering of a monochromatic source of photons is of interest (Raman) rather than absorption of a polychromatic beam (UV–Vis absorption), means that multiple optical arrangements must be accommodated in a multimode spectrometer. How these arrangements have been resolved in a manner compatible with macromolecular X-ray diffraction and then integrated into the beamline environment at X10SA of the Swiss Light Source (SLS) is described below.

## 2. Materials and methods

### 2.1. Experimental design

*In situ* UV–Vis absorption spectroscopy has previously been successfully implemented at the SLS protein crystallography beamlines using an off-axis geometry (Beitlich *et al.*, 2007 ▶). This arrangement resulted in several experimental limitations, some of which are common to most off-axis spectrometers currently in use. An off-axis arrangement greatly crowds the sample environment as, in addition to standard beamline components such as the collimator, alignment camera and illumination, two objective optics and an arc mount must be accommodated. While crowding can be alleviated to an extent by the use of long-working-distance objectives (McGeehan *et al.*, 2009 ▶), spatial restrictions mean that it is impractical to permanently install such a set-up at a synchrotron beamline. The objectives must therefore be reinstalled and aligned both with respect to each other and the beamline before each spectroscopy run, frequently a time-consuming procedure. An off-axis geometry also results in difficulties in ensuring the same sample volume is probed by X-rays and spectroscopy, and, as a consequence of the sample geometry, is more prone to spectral artefacts caused by reflections of the probing light.

The use of an on-axis geometry circumvents these drawbacks and allows the instrument to be permanently installed at the beamline. This avoids the time-consuming alignment procedures previously associated with spectroscopic experiments and raises the possibility of *in situ* spectroscopy becoming as commonplace as taking a fluorescence scan or changing the wavelength for MAD data collection. Co-axial simultaneous micro-Raman and synchrotron microdiffraction at ID13 of the ESRF has already been introduced as a powerful tool (Davies *et al.*, 2005 ▶), and here we detail the development of a novel solution for on-axis spectroscopy at beamline X10SA of the Swiss Light Source (Pohl *et al.*, 2006 ▶).

**Figure 2:**
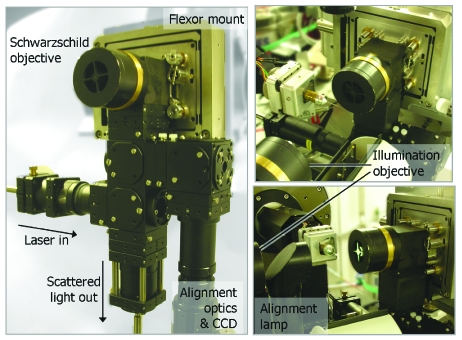
Left: the SLS multimode spectrometer mounted off-line, highlighting the extent of the system and arrangement employed. Top and bottom right: the spectrometer installed at X10SA. In both cases the illumination optic is shown in the far position for sample alignment. The box containing the scintillator, beam-defining apertures and collimator can be seen below the sample position. The cryostream has been removed for clarity.

The on-axis geometry of the SLS multimode microspectrophotometer (SLS-MS) (shown in Fig. 2 ▶) is achieved with a design analogous to that of existing on-axis microscopes, *i.e.* with a drilled objective mounted on the exposure box. The majority of on-axis microscopes utilize a drilled objective followed by a drilled mirror to deflect light away from the X-ray axis towards further alignment optics and a CCD detector mounted below the beamline.

**Figure 3:**
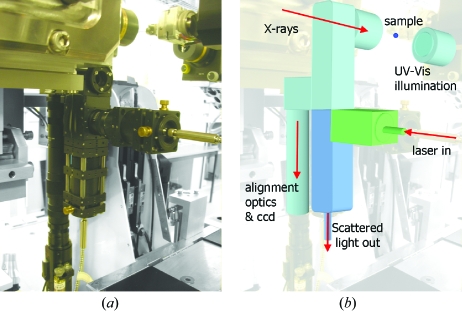
The branched nature of the SLS multimode spectrometer. The alignment branch (aqua) delivers light *via* infinity focused zoom optics to a CCD camera. The spectroscopy branch further divides into a branch for delivery of laser light for Raman spectroscopy (green) and a branch for collection of scattered light for all spectroscopies (blue).

For the SLS-MS, a high-magnification reflective Schwarz­schild objective (Newport, 15× magnification, *f* = 13 mm, numerical aperture 0.4) is used. This has the advantage of freeing up a large amount of space around the sample environment as the same objective can be used for both sample alignment and spectroscopy. Reflective objectives combine several desirable characteristics for both sample alignment and spectroscopy, including zero chromatic aberration, a high laser power threshold and a long working distance. Light collected by the reflective objective is reflected in a direction 90° below the X-ray axis to the branched SLS-MS (Fig. 3 ▶).

The branched design of the SLS-MS is shown in Fig. 3 ▶, with the alignment and spectroscopy branches highlighted in aqua and blue/green, respectively, in Fig. 3(*b*) ▶. In order to accommodate the multiple optical arrangements required by UV–Vis absorption and Raman spectroscopies, the spectroscopy branch is divided into two. For UV–Vis absorption spectroscopy, absorption of a polychromatic beam passing through the sample is of interest. Therefore an illuminating objective is required and only the collection path (blue) of the spectroscopy branch is utilized. For Raman spectroscopy, inelastic scattering of a monochromatic light source is of interest and a 180° scattering geometry is used. In this case an illuminating objective is not required and both the illumination (green) and collection (blue) paths of the spectroscopy branch are used (Fig. 3*b* ▶).

The illumination objective required for UV–Vis absorption spectroscopy is provided by a second Schwarzschild objective mounted on a motorized stage [shown in Figs. 2*b* (right) ▶ and 3 ▶]. This can be driven between three positions: (i) spectroscopic data collection; (ii) sample mounting and alignment; (iii) X-ray data collection. The reproducibility of the movement of this stage will be discussed in the following section. The unequal splitting between both spectroscopic sub-branches and the alignment and spectroscopy branches is achieved *via* the use of pellicle beam-splitters. These divide light between the branches in the ratio 1:12 with the extremely thin (∼2 µm) pellicle membranes eliminating refraction-based errors and ghost images.

The SLS Raman probe is a Kr^+^ gas laser (Coherent Innova 300C) allowing a range of wavelengths to be accessed;^1^ currently laser clean-up and holographic notch filter sets are available for the 413.1, 647.1 and 752.5 nm lines of the laser. In order to change the lasing wavelength, the laser mirrors must be exchanged and re-aligned. This procedure takes ∼30 min, with full laser powers achievable after a further 30 min as the laser warms up. Laser powers of the order of 10, 20 and 10 mW at the sample position are attainable at 413.1, 647.1 and 752.5 nm, respectively. As outlined above, this range of wavelengths allows a wide range of resonant modes to be accessed, while at the two longer wavelengths protein fluorescence is greatly reduced allowing these lines to be used for non-resonance Raman spectroscopy.

Both Raman and UV–Vis absorption spectra are recorded using an Andor 303i Czerny–Turner spectrograph and a Newton electron multiplying CCD (Andor technology). Two grating sets optimized for Raman data collection in the UV (413.1 nm Kr^+^ line) and red to near below-red (647.1 and 752.5 nm Kr^+^ lines) can be mounted within the spectrograph; for each grating set three line-spacings can be used allowing either ‘global’ spectra to be collected or a small region of interest to be investigated. Both the spectrograph and Kr^+^ gas laser are located in an optical hutch adjacent to the beamline allowing on-line experiments to make use of pre-aligned/optimized optical arrangements, and off-line experiments to operate independently of the beamline. Light is coupled between the spectroscopy hutch and beamline by means of 20 m optical fibres; for off-line experiments these can be replaced by the corresponding 2 m fibres. The demagnification ratio between a 50 µm fibre and the focal spot diameter is 0.69. The SLS-MS is not yet currently permanently installed at the beamline and so can be used for both on- and off-line measurements. Once the optical arrangements and design are finalized, and the system is permanently installed, all the components are available for constructing a duplicate off-line system.

## 3. Results

### 3.1. Optical and X-ray transmission properties of a drilled Schwarzschild objective

**Figure 4:**
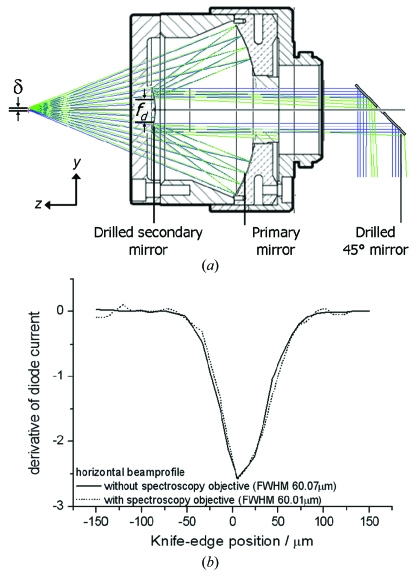
The effect of drilling on the optical and X-ray transmission properties of the Schwarzschild objective. (*a*) Ray traces calculated using the ray-tracing software *Zemax* (http://www.zemax.com/) from the focal point (blue) and a point a distance δ off-axis (green) through the system are shown. Translation of a knife-edge across the X-ray beam allows the profile to be determined with and without the spectroscopy objective in place; no change is observed in either (*b*) the horizontal or vertical (data not shown) directions.

The drilling of a 1 mm-diameter hole in the secondary mirror does not degrade the optical properties of the objective since the occluded region of this mirror is 4.6 mm in diameter. This free diameter, *f*_d_, is shown in Fig. 4(*a*) ▶. Of potentially greater impact on the light throughput of the system is the hole in the 45° mirror. This is illustrated by ray-tracing the light path from two points in the focal plane through the system: the focal point and a point a distance δ off-axis (shown in blue and green, respectively, in Fig. 4*a* ▶). Light from the focal point does not fall on this hole, although it is possible for light from the off-axis point to fall there, with the amount of light ‘lost’ increasing as a function of δ. If a value of 0.6 mm is taken for δ, corresponding to the full field of view of the objective, then the intensity of light lost is less than 2%. Holes of diameter 1 mm are large enough to allow X-rays to pass through the system even when the flexor mount is adjusted. This is illustrated in Fig. 4(*b*) ▶; in this case a knife-edge was scanned across the sample position with either the Schwarz­schild objective or normal on-axis viewing system lens mounted.

The current recorded by a diode placed behind the knife-edge allowed the X-ray beam profile to be determined in the horizontal and vertical (data not shown) in both cases. No change in the beam properties was observed with the Schwarzschild objective mounted, even in the case of a de­focused X-ray beam.

### 3.2. Alignment of optical and X-ray axes

**Figure 5:**
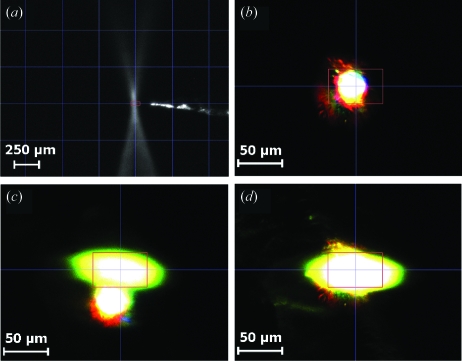
(*a*) Alignment of the focal point of the xenon lamp to the focal plane of the alignment camera using a camera mounted directly above the beamline and a pin at the sample position; the focus of the Schwarzschild objective can be clearly seen. The placement of a scintillator at the sample position allows the sample alignment camera to be used for alignment of the X-ray and optical axes: (*b*) X-ray shutter closed, optical shutter open; (*c*) both X-ray and optical shutters open with objective mis-aligned; (*d*) both X-ray and optical shutter open with the position of the yaw of the objective mount adjusted to maximize overlap of the X-ray and optical beams at the sample position.

The use of the same on-axis objective for sample alignment and spectroscopic data collection allows unambiguous alignment of the X-ray and visible optical axes. In order to achieve this, the flexor-mounted Schwarzschild objective is translated in *z* (see Fig. 4*a* ▶ for definition of axes) so that the centre of rotation of the sample is at the working distance of the objective. This can be achieved easily by use of a camera mounted above the beamline, and its view of the focusing of the xenon light by the Schwarzschild objective is shown in Fig. 5(*a*) ▶; the presence of the cryostream allows the visible light to be observed. Placement of a scintillator at the sample position then allows the X-ray and optical axes to be made coincident at the sample position. This is achieved by adjustment of the ‘pitch’ and ‘yaw’ of the flexor mount of the collection optics (Figs. 5*b*–5*d* ▶). The UV–Vis illumination Schwarzschild objective can be translated in *x*, *y* and *z* using its motorized stage and multi-axis fibre mount (Newport) which allows pitch and yaw adjustment to the fibre input. The focal spot of this objective is aligned to the centre of rotation of the sample by use of a 12.5 µm pinhole placed at the sample position through which the intensity of transmitted light at the spectrometer is maximized.

The focal plane of the alignment system can be fine-tuned and made exactly coincident with the laser/visible-light focal spot by means of a Kepler-type arrangement of two lenses in the alignment branch of the spectrometer (F. Schwarz, personal communication). This arrangement also allows the field of view to be matched to the size of the alignment CCD. Owing to the high magnification (15×) of the objectives, the field of view is limited. The full field of view is 1.2 mm, with uniform illumination possible over ∼600 × 400 µm for sample alignment, and we have found this area to be sufficient in the case where standardized pins are used throughout an experimental run. In the case where non-standard pins are used, coarse sample alignment using the camera mounted above the beamline has been integrated into the sample alignment graphical user interface to complement on-axis alignment.

This use of the same on-axis optic for sample alignment and spectroscopy allows simple and unambiguous alignment of the X-ray and visible-light axes ensuring the same sample volume is always probed by both diffraction and spectroscopy. As the scintillator is mounted on a motorized stage (Fig. 2*b* ▶) and can be easily moved to the sample position, this alignment can be checked at intervals throughout the experiment. The on-axis geometry also means that the optics can remain in place between experiments, eliminating the time-consuming alignment currently associated with spectroscopy at synchrotron sources. Tests of the reproducibility of the UV–Vis illumination objective position when driven between its three positions (spectroscopy, sample alignment and mounting, X-ray data collection) showed less than 3% change in transmitted intensity of the Xe lamp over ten iterations.

### 3.3. UV–Vis absorption spectroscopy

**Figure 6:**
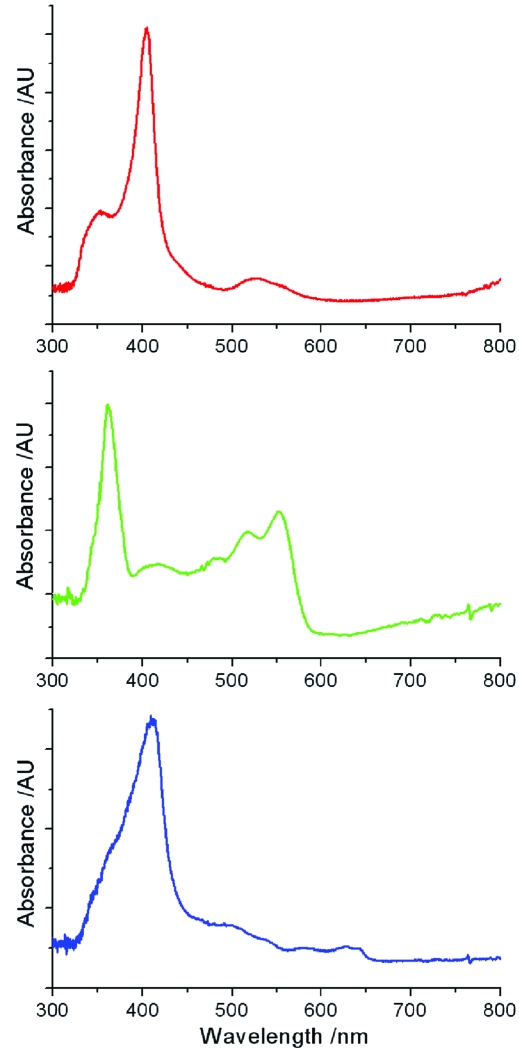
UV–Vis absorption spectra of cryocooled (100 K) cytochrome C thin film (top); vitamin B12 crystal (∼10 × 60 × 150 µm) (middle); and met myoglobin (metMb) crystal (∼10 × 80 × 150 µm) (bottom), crystallized as previously described (Beitlich *et al.*, 2007 ▶), taken using the on-axis spectrometer. The discontinuities observed at 470 and 765 nm are due to intense peaks in the xenon lamp spectra at these wavelengths. Data were collected with accumulation times of 10 × 100 µs, 5 × 20 µs and 4 × 0.8 ms, respectively, using a 10 µm slit width and a grating with 150 lines mm^−1^ in all cases.

Test single-crystal UV–Vis absorption spectra have been recorded for a variety of systems (Fig. 6 ▶). Owing to the transmission properties of the focusing objectives coupling light between the spectrometer and optical fibres, the available UV–Vis absorption range is approximately 325 nm to 850 nm. The transmission of the system is approximately linear between these wavelengths, and is ∼18%. Longer wavelengths are accessible but intense peaks of the xenon lamp in that region make simultaneous collection of short- and long-wavelength data difficult, though neutral density and coloured filters can be automatically introduced to the light path to alleviate this.

The Andor Shamrock 303i spectrograph is equipped with motorized adjustable slits (10 µm–1.2 mm) and the exposure times can vary from a minimum of 20 µs to considerably longer exposure times if required. This flexible arrangement allows matching of the data acquisition parameters to the optical properties of the experimental system.

**Figure 7:**
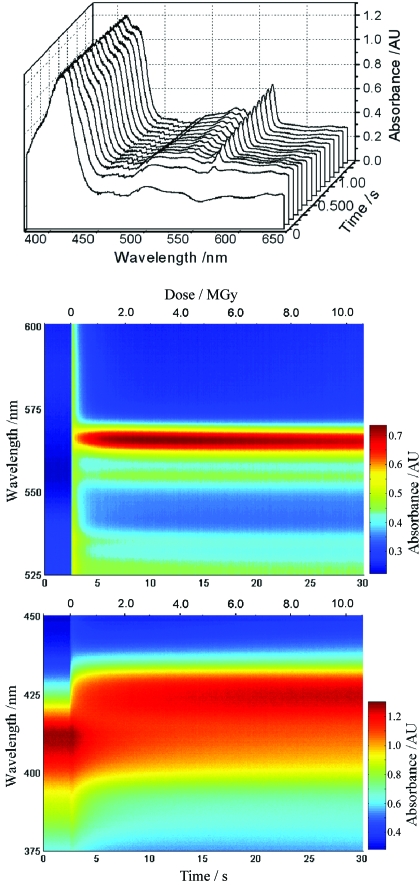
UV–Vis absorption spectra of a metMb crystal (∼10 × 90 × 120 µm) highlighting X-ray-induced changes at 100 K. Top: interval line plot, with a spectrum plotted every 0.1 s. Middle: contour plot showing the evolution of bands at 555 and 565 nm. Bottom: contour plot showing red shift of the 410 nm Soret peak; the time (*t* ≃ 2 s) at which the X-ray shutter is opened can clearly be seen by a global intensity increase as observed in other systems (Dubnovitsky *et al.*, 2005 ▶).

The SLS-MS has been used to investigate the sensitivity of two systems to radiation damage during X-ray exposure. The heme group of myoglobin is known to be sensitive to reduction by X-rays (Beitlich *et al.*, 2007 ▶). A myoglobin single crystal was mounted at beamline X10SA and UV–Vis absorption spectra were recorded during X-ray exposure without rotation of the crystal (Fig. 7 ▶). Four accumulations of 0.8 ms exposures were recorded every 0.1 s. During data collection the crystal was not rotated in order to avoid spectral changes owing to variation in the crystal orientation, apparent changes in the crystal thickness, and the effect of the loop entering the light path (Wilmot *et al.*, 2002 ▶). As single-crystal spectra are anisotropic, crystals were pre-orientated to a ‘sweet spot’ where the anisotropic spectra best resembled the isotropic solution spectra. Rapid reduction occurs, as evidenced by a red shift in the Soret band and appearance of characteristic peaks at 550–560 nm (Fig. 7 ▶), and is essentially complete within 8 s of initial X-ray exposure corresponding to an absorbed dose of ∼3 MGy [absorbed dose calculated using *RADDOSE* (Murray *et al.*, 2004 ▶)].

**Figure 8:**
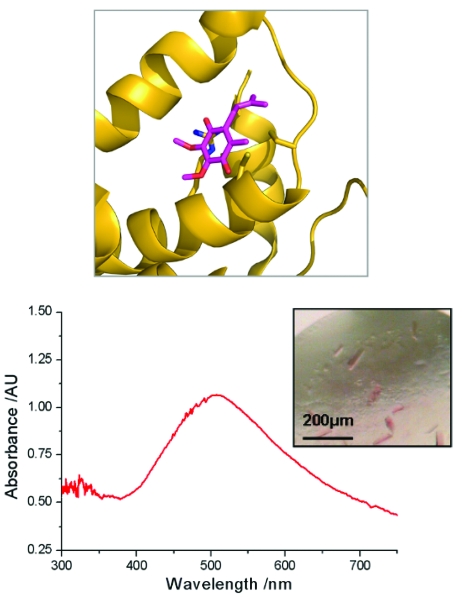
Top: a charge transfer interaction between wild-type DsbB and ubiquinone induces a pink colouration in the quinone group. Inset bottom: *in situ* UV–Vis absorption spectroscopy of DsbAB crystals confirm the presence of the charge transfer interaction in the form of (bottom) a broad absorption peak centred at 510 nm.

Radiation effects were investigated in crystals of a second system, a ubiquinone binding *E. coli* membrane protein, DsbB, involved in disulfide bond formation (Malojcic *et al.*, 2008 ▶). DsbB is known to induce a red shift in ubiquinone upon binding (Inaba *et al.*, 2004 ▶) and a characteristic red-shifted visible peak appears upon ubiquinone binding within the DsbA–DsbB–ubiquinone (DsbAB-Q8) complex. Unlike heme-containing proteins in which the Soret band has a large molar extinction coefficient, this peak has a small extinction coefficient (∼4750 cm^−1^ *M* ^−1^)*.* However, despite the low intensity of this peak, it can be clearly observed in the SLS-MS with only 4 × 20 µs exposures (10 µm slits) (Fig. 8 ▶).

Fig. 8 ▶ also highlights the value of the complementary information that UV–Vis absorption spectroscopy can provide. Owing to the limited diffracting power of DsbAB-Q8 crystals (3.7 Å for the best crystals in the most favourable orientation), it is not possible to identify unambiguously the presence of the charge transfer interaction in the crystalline state *via* X-ray diffraction alone (Malojcic *et al.*, 2008 ▶). *In situ* UV–Vis absorption spectra allow spectroscopic confirmation of the presence of the interaction within the crystal, aiding interpretation of crystallographic data. In contrast to the example of myoglobin above, the UV–Vis absorption spectra of DsbAB-Q8 do not change as a function of X-ray dose, revealing the interaction to be radiation insensitive over the periods required to collect a complete crystallographic data set. The dynamic range of the instrument is highlighted by its capability to measure both the myoglobin Soret band reported above and the 510 nm DsbAB-Q8 quinone signal.

### 3.4. Raman spectroscopy

**Figure 9:**
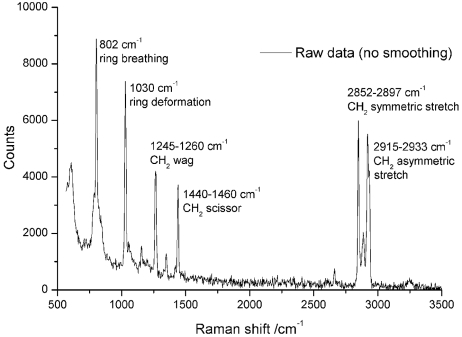
Raman spectra of a cyclohexane thin film collected using the 647.1 nm laser line for excitation. Spectra were collected with 4 × 5 s exposures using a grating with 300 lines mm^−1^, allowing the entire spectrum to be collected simultaneously. Major spectral features are labelled.

The Raman capabilities of the SLS-MS are presently undergoing commissioning. We show here initial data from a small molecule standard (cylcohexane, Fig. 9 ▶). Clear Raman peaks are visible, even for relatively short exposure times. Spectra were collected using the 647.1 nm line of the Kr^+^ laser with 4 × 5 s exposures, a spectrograph grating with 300 lines mm^−1^ and a slit width of 40 µm. The calculated spectral resolution of the system at this laser wavelength with this grating is ∼10 cm^−1^. The data have been left unsmoothed or baseline corrected to highlight the quality of data obtained, and major spectral features are labelled. The results using these parameters are extremely encouraging when compared with other single-crystal Raman systems.

### 3.5. X-ray data collection

Measurements at the sample position confirm that the reflecting objective does not reduce the available X-ray flux, with knife-edge scans and diode measurements showing identical beam profiles and fluxes with and without the spectrometer mounted (Fig. 4*b* ▶). Diffraction tests with the microspectrophotometer mounted show that there is no effect on diffraction data quality (data not shown). As the illumination objective must be lowered in order to collect diffraction data, simultaneous UV–Vis absorption and X-ray diffraction data cannot be collected; this, however, is not a limitation as good quality UV–Vis absorption data can usually only be collected at a particular crystal orientation (Wilmot *et al.*, 2002 ▶; Pearson *et al.*, 2007 ▶). The 180° scattering geometry utilized for Raman spectroscopy means that simultaneous (resonance) Raman and X-ray diffraction experiments are possible using the SLS-MS since only a single spectroscopy objective is required, though it is anticipated that Raman and X-ray data collection will rather be interleaved for optimal crystal orientation during spectroscopic data collection.

## 4. Discussion

The SLS-MS is the first on-axis single-crystal spectrometer at a synchrotron macromolecular crystallography beamline. Its design allows it to be permanently installed at the beamline, removing the need for time-consuming installation and alignment associated with non-permanent off-axis systems. Designed as an integral part of the beamline, it has an uncrowded sample environment that allows other beamline components such as the robotic sample changer and fluorescence detector access to the sample, and also facilitating manual crystal mounting.

The facility to have both X-ray and spectroscopy probing the same sample volume in the SLS-MS circumvents a persistent problem with off-axis UV–Vis absorption single-crystal spectroscopy instruments. Unless the X-ray beam size is greater than or equal to the crystal size, an off-axis spectrometer will probe both X-ray irradiated and unirradiated volumes of the crystal. This is a major problem when attempting to follow changes resulting from radiation damage by UV–Vis absorption spectroscopy, although less of an issue for Raman spectroscopy if the spectrometer is correctly focused. The degree of change in the X-ray-illuminated volume may therefore be mis-estimated by off-axis UV–Vis absorption spectroscopy, resulting in incorrect decisions concerning the permissible dose a crystal can receive before the final electron density map is dominated by a radiation-damaged state. A second advantage related to the on-axis geometry is the ability to perform photoactivation experiments using the standard beamline set-up. The SLS-MS on-axis arrangement and availability of a range of laser excitation wavelengths allows laser excitation of the sample volume to be probed by the X-rays, as well as permitting simultaneous spectroscopic characterization of the efficiency of laser excitation. In addition, linked gating of the laser and X-ray shutters is possible. This, combined with an eight-fold increase in the laser power at the sample position (by switching of the laser fibre input to the spectroscopy branch of the spectrometer) if photoactivation is the sole aim of the experiment, makes the SLS-MS particularly well suited to this type of experiment. For thick samples and/or samples with high extinction coefficients, classical orthogonal absorption experiments may provide more uniform sample excitation/illumination owing to the limited penetration of the laser beam (Anderson *et al.*, 2004 ▶; Schotte *et al.*, 2003 ▶, 2004 ▶).

The SLS-MS is now in the final stages of commissioning before permanent installation at beamline X10SA. The current design, based on modular components, provides great flexibility for optimization and variation of the optical arrangement. It is anticipated that, once the optical arrangement is finalized, the body of the spectrometer will be machined as a single component which will give additional stability during regular user operation. Although the UV–Vis spectroscopy set-up is now stable and ‘user ready’, the Raman spectroscopy requires further commissioning to optimize it for use with protein crystals. Preliminary studies have shown that, unlike commercially available Raman probes used in other systems, considerable inelastic fibre scattering effects as well as protein fluorescence obscure much of the low wavenumber range (<1000 cm^−1^). Additional optical components alleviating these effects are currently being commissioned for testing, as is improved optical fibre coupling to further increase the light throughput, sensitivity and spectral range of the spectrometer. The combination of the SLS-MS on-axis and the 180° Raman scattering geometries permits permanent provision of simultaneous collection of Raman spectroscopy and X-ray diffraction data at X10SA. Beamline software for both ease of use and avoidance of potential collisions of the UV–Vis absorption illumination optic with beamline components (for example beamstop, sample illumination lamp and detector) is currently being integrated into the experimental arrangement. Also under investigation is the use of a kappa geometry to allow the measurement of Raman bands in different crystal positions in order to derive information about the orientation of specific vibrations with respect to the crystal axes.

The 180° scattering geometry used by the SLS-MS for Raman spectroscopy is also well suited to other spectroscopies. Collection of both fluorescence (using either the available xenon lamp or Kr^+^ laser for excitation) and X-ray excited optical luminescence (XEOL) spectra are possible without further modification to the instrument design. Initial XEOL experiments have been carried out (data not shown) indicating that the spectrometer design is compatible with fluorescence-type experiments.

The development of the instrument described here allows a spectrometer capable of UV–Vis absorption and (resonance) Raman spectroscopy to be permanently integrated into the beamline environment, making the on-axis SLS multimode spectrometer an extremely attractive tool for obtaining complementary spectroscopic information and pursuing kinetic crystallography at macromolecular crystallography beamlines.

## Footnotes

1 For a full list of Kr^+^ lines, see http://facs.scripps.edu/Lasers.html.
